# The effect of misoprostol on the removal of endometrial polyps: A pilot clinical trial

**DOI:** 10.18502/ijrm.v20i6.11441

**Published:** 2022-07-06

**Authors:** Abbas Aflatoonian, Zohreh Pezeshkpour, Yasamin Mehrolhasani, Nasim Tabibnejhad

**Affiliations:** Research and Clinical Center for Infertility, Yazd Reproductive Sciences Institute, Shahid Sadoughi University of Medical Sciences. Yazd, Iran.

**Keywords:** Endometrial polyps, Misoprostol, Saline infusion sonohysterography, Transvaginal color Doppler, Infertility.

## Abstract

**Background:**

Endometrial polyps are one of the most common gynecological disorders with a high frequency among infertile women. Hysteroscopic polypectomy remains the gold standard for the treatment of endometrial polyps. As alternative treatments, few drugs have been evaluated to date.

**Objective:**

To investigate the possible effect of misoprostol on the elimination of endometrial polyps.

**Materials and Methods:**

In this clinical trial we examined 30 infertile women whose endometrial polyps were confirmed by transvaginal ultrasound with saline injection. All women were administered 400 mg of misoprostol: 200 mg orally and 200 vaginally. 8 hr later, sonography with saline injection was performed again and all women were examined for the presence or absence of endometrial polyps. Finally, the diagnosis was confirmed for all women using hysteroscopy. The main outcome of this study was the elimination of endometrial polyps after misoprostol administration.

**Results:**

The average size of the endometrial polyps was 14.33 ± 4.26 mm, with a range of 7-22 mm. After misoprostol administration, in 12 out of the 30 women who had shown endometrial polyps in the initial examination, no polyp was found. At follow-up it was found that the smallest endometrial polyp that had been eliminated was 8 mm and the largest was 22 mm.

**Conclusion:**

The findings of our study revealed that misoprostol can remove up to 40% of endometrial polyps. This drug has the potential to be used as a safe and low-cost first-line treatment before performing hysteroscopic polypectomy.

## 1. Introduction 

Uterine polyps are one of the most common gynecological disorders and they often present as abnormal uterine bleeding (1). Endometrial polyps are caused by the abnormal growth of glands, stroma or blood vessels, and they protrude from the surface of the endometrium into the uterine cavity (2, 3). Risk factors for endometrial polyps include aging, obesity, hypertension, and the use of tamoxifen (4, 5). In clinics, their prevalence is estimated to be 7.8-34.9% but they are even more common in infertile women (3, 6). Moreover, an increase in the pregnancy rate of 23-65% has been reported after polypectomy (7-9). The molecular mechanism of how the polyps trigger infertility is related to a disturbance in endometrial receptivity (10, 11).

On transvaginal ultrasounds, endometrial polyps are seen as hyperechoic areas with a regular environment surrounded by a thin hyperechoic halo inside the uterine cavity (12). Studies have shown that the addition of the vascular Doppler augments the capacity of the vaginal ultrasound in the diagnosis of endometrial polyps (13). Observation of a single feeding vessel in Doppler color flow ultrasound is a typical view of endometrial polyps (14). Indeed, ultrasound with saline injection, when accompanied by the sight of this single feeding vessel, is known to be a key method in the diagnosis of endometrial polyps in premenopausal women (15). Some researchers have considered negative results of sonohysterography as being sufficient in ruling out intrauterine anomalies (16). Nevertheless, other studies have reported that saline infusion sonohysterography (SIS) and hysteroscopy are not significantly different in their effectiveness in diagnosing endometrial polyps (17-19).

Regarding endometrial polyp treatment, hysteroscopic polypectomy remains the gold standard for endometrial polyp removal (13, 20, 21). However, hysteroscopic polypectomy commonly results in complications such as infection, hemorrhage, pelvic inflammatory diseases, uterine rupture or cervical injury, and the need for the use of gas or fluid to dilate the uterus should also be taken into account (2). Only a few alternative treatments have been studied so far, including the use of the intrauterine device producing Levonorgestrol, oral contraceptives, and gonadotropin-releasing hormone (22-24).

At Yazd Reproductive Sciences Institute, we routinely use misoprostol (Cytotec, 200 Mcg, Pfizer, Germany) to prepare the cervix before hysteroscopic polypectomy in infertile women for whom endometrial polyps have been detected on vaginal ultrasound. Interestingly, we have noticed that in these cases there have been no signs of the polyps when the women underwent hysteroscopic polypectomy. Therefore, we aimed to investigate the possible effect of misoprostol on the elimination of endometrial polyps.

## 2. Materials and Methods

In this pilot clinical trial conducted from July to November 2020 at the Yazd Reproductive Sciences Institute, Yazd, Iran 30 infertile women aged 18-45 yr were studied. Infertility was defined as the inability to achieve pregnancy after 1 yr of regular intercourse without using any contraceptives (1). The presence of endometrial polyps was confirmed in all women by sonography with saline injection. Transvaginal ultrasound with color Doppler (TVCD) needed to be performed in some cases. Women with untreated uterine malformations, pelvic inflammatory diseases, or a history of sensitivity to prostaglandin analogues were excluded from the research. The process of ultrasound with saline injection, misoprostol, and finally, hysteroscopy were fully explained to all of the women.

The women underwent SIS in the proliferative phase. 20 min before SIS, 500 mg of oral azithromycin tablets (Shafa Pharmaceutical & Hygienic Co., Iran) and a diclofenac suppository of 100 mg (Darou Pakhsh Pharmaceutical Mfg Co., Iran) were used in all the women, and sonohysterography with an intrauterine injection of 20-25 cc of normal saline was performed. TVCD (Phillips model Affiniti 70 W, The Netherlands) was performed for some cases by an experienced and trained physician to confirm the diagnosis. The endometrial polyp was defined as a localized hyper echo lesion in color Doppler characterized by a smooth and well-defined border with a feeding vessel. If the endometrial polyp was confirmed at this stage, the size of the polyp was also determined and the women's characteristics including age, body mass index (BMI), and the type of infertility, being either primary or secondary, were recorded.

All women were administered 400 mg of misoprostol (Cytotec, 200 Mcg, Pfizer, Germany), 200 mg orally and 200 vaginally, 48 hr after the confirmation of endometrial polyp diagnosis. 8 hr later, all women were examined for the presence or absence of endometrial polyps. Finally, all women underwent hysteroscopy for confirmation of the diagnosis and an endometrial biopsy was taken. Moreover, in the 18 cases in which endometrial polyps remained after misoprostol administration, polypectomy was performed through a resectoscope.

### Ethical considerations

All of the women were fully explained about process of ultrasound with saline injection, misoprostol, and finally hysteroscopy. They all signed the informed consent form. The study protocol was approved by the Ethics Committee of Yazd Reproductive Sciences Institute, Shahid Sadoughi University of Medical Sciences, Yazd, Iran (Code: IR.SSU.RSI.REC.1399.010).

## 3. Results

**Figure 1:**
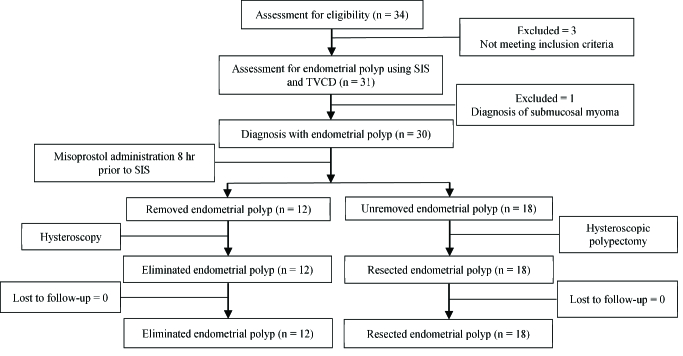
Flow-chart of the endometrial polyp diagnosis and treatment, TVCD: Transvaginal color doppler, SIS: Saline infusion sonohysterography.

Initially, 34 women were enrolled in the study. 3 of these women did not meet the inclusion criteria. Therefore, 31 women were assessed for endometrial polyps through SIS and in some cases SIS plus TVCD. 1 woman was excluded from the study due to a diagnosis of submucosal myoma. Finally, 30 women with a diagnosis of endometrial polyps received misoprostol (Figure 1). The demographic and clinical features of all of the participants are listed in table I. The mean age of the women was 34.00 ± 4.79 yr and their mean BMI was 27.31 ± 3.74. In total, 23 women (76.7%) had a BMI ≥ 25, 17 women (56.7%) had secondary infertility and 13 (43.3%) reported a history of abortion. The average size of the endometrial polyps was 14.33 ± 4.26 mm, the smallest of which was 7 mm and the largest was 22 mm (Table I).

**Figure 2:**
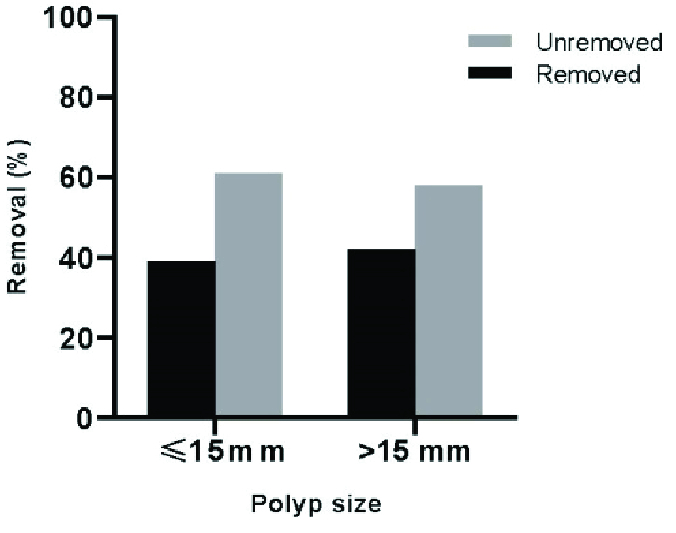
Endometrial polyp removal rate based on the polyp size.

After misoprostol administration, in 12 out of the 30 women who had shown endometrial polyps in the initial examination, no polyp was found; this was identified through both SIS follow-up and hysteroscopy. At the follow-up it was found that the smallest endometrial polyp that had been eliminated was 8 mm and the largest was 22 mm. There was no statistically significant correlation between the polyp size and polyp elimination after misoprostol administration (p = 0.87). As is shown in figure 2, 41.7% of the endometrial polyps larger than 15 mm and 38.9% of those ≤ 15 mm were eliminated after misoprostol administration. No serious side effects for misoprostol were reported except for pelvic pain in some cases. The most common abnormal pathology associated with the endometrial polyps was proliferative disorder which was observed in 5 cases (35.0%), and 1 case (3.3%) showed an atypical myofibromatous polyp. In addition, all of the results obtained in the follow-up of the patients by the SIS and TVCD methods in terms of the presence or absence of endometrial polyps were consistent with the hysteroscopy results.

**Table 1:** The mean and standard deviation of participants' age, BMI and polyp size (n = 30)


	**Mean ± standard deviation**
**Age (yr)**	34.00 ± 4.79
**BMI (kg/m^2^)**	27.31 ± 3.74
**Polyp size (mm)**	14.33 ± 4.26
BMI: Body mass index	

## 4. Discussion

As far as we know, our study is the first to investigate the effect of misoprostol on the removal of endometrial polyps in infertile women. In our study, misoprostol had a therapeutic effect on 40% of polyps in triggering their removal from the uterus.

Misoprostol is a synthetic analogue of prostaglandin E1, which has several applications in obstetrics and gynecology (25). The advantage of misoprostol over other prostaglandin analogues is its long half-life, availability, lack of a need for refrigeration, and low cost (26). In our study, a dose of 400 micrograms of misoprostol was used, which is widely deployed in obstetric patients, and no serious side effects have been reported with this dose (27). In addition, studies have demonstrated that doses of 200, 400 and 1000 mg of misoprostol taken vaginally or orally 9-12 hr before hysteroscopy are effective in dilating the cervix in premenopausal women (28-30).

Wada-Hiraike and colleagues examined the effect of oral contraceptive pills on the removal of uterine polyps and concluded that stalkless polyps respond better to oral contraceptives than stalked polyps (24). Moreover, Chowdary and colleagues reported the beneficial effect of the intrauterine device Mirena in eliminating polyps in premenopausal women with heavy menstrual bleeding (31). However, based on the 2 mentioned studies, the treatment of polyps lasted an average of 80-90 days, and the use of these drugs for several months is hardly welcomed by infertile patients. Vercellini et al. displayed the beneficial effect of gonadotropin-releasing hormone agonist as an adjuvant polyp treatment before hysteroscopic resection; however, the common use of this drug is not appealing due to its side effects and cost (23).

One of the strengths of our study was its prospective aspect that applied a combination of several early diagnostic methods to confirm uterine polyps so as to minimize diagnostic error. In this study, all ultrasounds, including TVCD and SIS, were performed by a qualified physician to minimize the potential effect of operator error. Sensitivity, specificity, positive predictive value, and negative predictive value of vaginal ultrasound in the diagnosis of endometrial polyps have been shown to be 86%, 94%, 91% and 90%, respectively (32). Using TVCD, the sensitivity increases up to 97% (33). Although hysteroscopy and guided biopsy are standard diagnostic methods for endometrial polyps, they are invasive procedures and are hence unlikely suitable to be repeated at short intervals of time (1). Therefore, in order to better confirm endometrial polyps, in addition to ultrasound with saline injection as a safe procedure, we used TVCD which has been demonstrated as equally effective as hysteroscopy in several studies (17, 18).

In various studies, SIS, compared with hysteroscopy, has been identified as having a sensitivity of 58-100%, a specificity of 35-100%, a positive p-value of 70-100%, and a negative p-value of 83-100% (17, 34-37). Furthermore, because our patients were infertile, deploying SIS as a diagnostic method could not only confirm the presence of the polyp but also provide the possibility to examine the fallopian tubes for openness and evaluate the uterine cavity and other structures inside the pelvis (12, 18). Another strength of our study was that time was not wasted on unnecessarily providing the standard treatment for polyps (hysteroscopic polypectomy) for infertile women, and the women could also receive other appropriate infertility treatments as soon as possible after diagnosis. The main weakness of this study was the lack of a control group.

Celik et al. investigated the effect of misoprostol on uterine blood flow in patients with uterine myoma and observed a reduction in uterine artery blood flow following misoprostol administration. They proposed that vasoconstriction, and thus contraction of uterine muscles, could explain this effect of misoprostol. They suggested that misoprostol can likely be regarded as a treatment option for uterine myoma in the future (27). In our study, misoprostol could eliminate 40% of the endometrial polyps; however, our results showed that there was no statistically significant association between the polyp size and polyp removal. It has previously been indicated that endometrial polyps larger than 15 mm may be less likely to display spontaneous regression, and that polypectomy should be considered for symptomatic patients (3, 38). Nonetheless, in the current study, a high percentage of polyps larger than 15 mm (41.7%) were resolved by misoprostol without polypectomy. The vasoconstrictor effect of the drug on the artery feeding the polyp and thus the formation of uterine contractions might have triggered the polyp to be eliminated from the surface of the endometrium.

Our study was the first to investigate the effect of misoprostol on the removal of endometrial polyps. Further studies are thus recommended which include a control group and employ repeated high doses of this drug, to investigate its possible further efficacy and to see if it can be considered as a safe, low-cost, and first-line treatment alternative for uterine polyps.

## 5. Conclusion

In conclusion, the findings of our study revealed that misoprostol can remove up to 40% of endometrial polyps. This drug has the potential to be used as a safe and low-cost first-line treatment before performing hysteroscopic polypectomy.

##  Conflict of Interest

The authors declare that there is no conflict of interest.
