# Corrigendum to “The effect of misoprostol on the removal of endometrial polyps: A pilot clinical trial” [Int J Reprod BioMed 2022; 20: 461-468]

**DOI:** 10.18502/ijrm.v20i12.12570

**Published:** 2023-01-09

**Authors:** Abbas Aflatoonian, Zohreh Pezeshkpour, Yasamin Mehrolhasani, Nasim Tabibnejad

The authors have been informed of an error that occurred on page 461 in which the family name of the last author (*Nasim Tabibnejhad*) has been entered incorrectly, which should be corrected as: “Nasim Tabibnejad". On behalf of the author, the publisher wishes to apologize for this error. The online version of the article has been updated on 20 December 2022 and can be found at https://doi.org/10.18502/ijrm.v20i6.11441.

